# Supplementary material for: Observation of magnetic adatom-induced Majorana vortex and its hybridization with field-induced Majorana vortex in an iron-based superconductor
Source: Nat Commun. 2021 Mar 1;12:1348. doi: 10.1038/s41467-021-21646-x (PMC7921435; doi:10.1038/s41467-021-21646-x)
Supplement: Supplementary file 1 — Supplementary Information [file 41467_2021_21646_MOESM1_ESM.pdf]

Supplementary Information for

Observation of magnetic adatom-induced Majorana vortex and its hybridization with field-induced Majorana vortex in an iron-based superconductor

Peng Fan,[1,2,†] Fazhi Yang,[1,2,†] Guojian Qian,[1,2,†] Hui Chen,[1,2,3,†] Yu-Yang Zhang,[1,2,4] Geng Li,[1,2,4] Zihao Huang,[1,2] Yuqing Xing,[1,2] Lingyuan Kong,[1,2] Wenyao Liu,[1,2] Kun Jiang,[1,5] Chengmin Shen,[1,2,4] Shixuan Du,[1,2,4] John Schneeloch,[6] Ruidan Zhong,[6] Genda Gu,[6] Ziqiang Wang,[5,*] Hong Ding,[1,3,4,*] and Hong-Jun Gao[1,2,3,4,*]

[1]Beijing National Laboratory for Condensed Matter Physics and Institute of Physics, Chinese Academy of Sciences, Beijing 100190, China

[2]School of Physical Sciences, University of Chinese Academy of Sciences, Beijing 100190, China

[3]Songshan Lake Materials Laboratory, Dongguan, Guangdong 523808, China

[4]CAS Center for Excellence in Topological Quantum Computation, University of Chinese Academy of Sciences, Beijing 100190, China

[5]Department of Physics, Boston College, Chestnut Hill, Massachusetts 02467, USA

[6]Condensed Matter Physics and Materials Science Department, Brookhaven National Laboratory, Upton, New York 11973, USA

[†]These authors contributed equally to this work.

[*]Correspondence to: wangzi@bc.edu, dingh@iphy.ac.cn, hjgao@iphy.ac.cn

SUPPLEMENTARY NOTE 1: Characterizations of samples and Fe adatoms

Previous measurements of the vortex core states in $FeTe_{0.55}Se_{0.45}$ under an external magnetic field have indicated that there are both topological trivial and topological nontrivial regions on the surface[1]. Before Fe deposition, we scan the surface of $FeTe_{0.55}Se_{0.45}$ to ensure that there is no interstitial iron atom (Supplementary Fig. 1a). We can also detect MZMs in vortices induced by an external magnetic field as exemplified in Supplementary Fig. 1, which demonstrates that the sample surface has topological nontrivial regions. Then, we perform atomic Fe deposition and focus on Fe adatoms. An example of a single Fe adatom is shown in the atomic-resolution STM topography (Supplementary Fig. 2), where the C_4 symmetric site is highlighted by the vertices of the bright dashed grid. It can be seen clearly that this Fe adatom is located at the high-symmetry site. Away from the Fe adatom, the "higher" sites correspond to the Te atoms and the "lower" ones to the Se atoms. The line-profile of the Fe adatom yields a height of 1.61 Å and the peak width at half maximum of 1.5 nm (Supplementary Fig. 2b). In general, the adsorption sites of the Fe adatoms can be on or off the C_4 symmetry axis piercing vertically through the center of four Te/Se atoms and located at different heights to the surface. From the statistics of hundreds of measurements, we find that the height of deposited Fe adatoms varies from 1.1 Å to 2 Å as shown in the histogram (Supplementary Fig. 2c) with an average value of 1.56 Å that is higher than the typical height (~1 Å) of the interstitial Fe impurities[2] in $FeTe_{0.55}Se_{0.45}$.

SUPPLEMENTARY NOTE 2: Integer quantized in-gap states at type-I Fe adatoms

We show the analysis of three additional type-I Fe adatoms in Supplementary Fig. 3. Similar to the case presented in Fig. 2 of the main text, the zero-energy dI/dV maps (Supplementary Figs. 3a, d and g) and the dI/dV spectra along the line-cuts across the adatoms (Supplementary Figs. 3b, e and h) exhibit sharp ZBPs together with several pairs of in-gap states, which do not disperse spatially. Performing the analysis in Fig. 2 of the main text, we find that the energies of the in-gap states at each adatom follow a series of near-integer quantization, which can be seen from the energies of the peak positions and ratios displayed in Supplementary Figs. 3c, f and i. Similar integer quantization of the in-gap states has been observed in the magnetic field-induced vortices in $FeTe_{0.55}Se_{0.45}$[1], providing compelling evidence that these in-gap states are the topological CdGM vortex core states of the QAV[3] nucleated at type-I Fe adatoms on the superconducting TSS, and the ZBP corresponds to the MZM.

SUPPLEMENTARY NOTE 3: Manipulation of a single type-I Fe adatom

To study the role of the high-symmetry adsorption site of the type-I Fe adatoms, we manipulate the location of the Fe adatom characterized in Fig. 2 of the main text using the STM tip. We first move the Fe adatom off the C_4 symmetric center surrounded by four Te/Se atoms into a different location (Supplementary Fig. 4a). Then we measure the zero-energy dI/dV map and the dI/dV spectra cross the adatom site along a line-cut as shown in Supplementary Fig. 4b and 4c. The pattern in the zero-energy dI/dV map and the dI/dV spectra along the line-cut change significantly compared to Fig. 2b-c in the main text: the ZBP disappears and a pair of in-gap states emerge at non-zero energies. Subsequently, we heat the sample up to 15 K and observe that the Fe atom diffuses back to the original high-symmetry site Supplementary Fig. 4d. We then cool the sample back down to 0.4 K and perform again the dI/dV measurements. As can be seen in Supplementary Fig. 4e-f, the ZBP reappears and the zero-energy dI/dV map as well as the spectra along the line-cut recover the spatial distribution of the zero-mode and the conductance spectra of those before the manipulation (Fig. 2b-c in the main text). The results indicate that the high-symmetry site is necessary for the induced ZBP by the type-I Fe adatoms. Finally, we remove the Fe adatom using the STM tip (Supplementary Fig. 4g). The measured spectra show a hard superconducting gap in the same area, demonstrating that the in-gap states originate from the Fe adatom (Supplementary Fig. 4h-i).

SUPPLEMENTARY NOTE 4: YSR states and Zeeman splitting on type-II Fe adatoms

The high-resolution topographic image shows an isolated type-II adatom (Supplementary Fig. 5a). The dI/dV spectra along the red dashed line-cut in Supplementary Fig. 5a are displayed in the waterfall plot (Supplementary Fig. 5b) and the intensity plot (Supplementary Fig. 5c). In contrast to the type-I adatoms, the conductance exhibits predominantly a pair of in-gap peaks at nonzero energies without the ZBP. Applying an external magnetic field, we observe that the peaks shift to higher energies (Supplementary Fig. 5d) away from the Fermi level, consistent with a pair of spin-polarized YSR in-

gap states. The energy positions of the YSR states under different magnetic fields can be fitted by a linear Zeeman splitting with the g-factor of about 0.88 (Supplementary Fig. 5e).

Because the energies of the in-gap states are controlled by the exchange interaction strength, it is known that accidental near degeneracy of the YSR states close to zero-energy (the near-zero YSR states) can arise. The atomic resolution STM topography (Supplementary Fig. 6a) and the zero-energy dI/dV map (Supplementary Fig. 6b) show an example of such a type-II Fe adatom. The evolution of the dI/dV spectra along the red line-cut across the adatom (Supplementary Fig. 6c) and its intensity plot (Supplementary Fig. 6d) clearly reveal the near-zero energy bound states whose spectroscopic features are similar to the ZBP detected on the type-I Fe adatoms in the absence of an external magnetic field. However, when external magnetic field is applied, the accidental degeneracy is removed as the near-zero YSR states show apparent Zeeman splitting into the usual YSR states (Supplementary Fig. 6e). The energy splitting can be well fit by a linear function in the magnetic field with a g-factor about 0.5. These observations clearly demonstrate that one can distinguish the near-zero YSR states from the robust ZBP by applying external magnetic fields. The near-zero YSR states with Zeeman splitting account for about 6.4% of the Fe adatoms in our measurements.

SUPPLEMENTARY NOTE 5: Modulating YSR states with approaching STM tip at type-II Fe adatoms

The method of varying the tip-sample distance has been used to change the coupling between the adatoms and the surface. In recent works[4, 5], the exchange coupling between a magnetic impurity and a BCS superconductor (Pb) has been shown to be tunable and the quantum transition of the YSR states by approaching the STM tip at the impurity sites has been realized. We first check the effectiveness of this method in our system by approaching the tip at the type-II Fe adatom site shown in Supplementary Fig. 7a. We measure the tip-sample distance by the offset z in Supplementary Fig. 7b, which shows that reducing z leads to a monotonic increase in the tunnel barrier conductance G_N. Before reducing the tip-sample distance, i.e. at the distance offset $z = 0$ in Supplementary Fig. 7b, the particle-hole symmetric (in energy) YSR states have a larger spectral weight on the negative energy side, visible in the dI/dV spectra at the bottom in Supplementary Fig. 7c as well as in the intensity plot (Supplementary Fig. 7d) at the smallest tunnel barrier conductance G_N. As the STM tip is made to approach the adatom, the YSR states evolves with increasing G_N toward and cross zero energy to the superconducting gap

edge with the larger spectral weight shifted to be on the positive energy side (Supplementary Fig. 7c-d). This is consistent with the increasing of the exchange coupling between the Fe adatom and the superconducting surface that causes a quantum transition of the YSR states with the increasing tunnel-barrier conductance G_N. Note that the *z*-offset decreases smoothly with the barrier conductance G_N (Supplementary Fig. 7b), indicating no change of the tip and the Fe atom during the approaching process.

SUPPLEMENTARY NOTE 6: Robustness of the ZBP under approaching STM tip at type-I Fe adatoms

We carry out tests on the robustness of the ZBP induced by type-I Fe adatoms under an approaching STM tip. The normalized STM intensity plots in zero-field and under an external magnetic field of 6T are shown in Supplementary Figs. 8c and 8d as a function of the tunnel barrier conductance G_N. The ZBP at the type-I Fe adatom is remarkably robust and does not shift or split with increasing G_N. The robustness of the ZBP at type-I Fe adatoms against increasing exchange interaction is illustrated in the schematic diagram in Supplementary Fig. 8b, which should be contrasted to the transition from the YSR states to the ZBP observed at the type-II Fe adatoms illustrated in Fig. 3b in the main text.

SUPPLEMENTARY NOTE 7: Replicable transition between YSR states and a vortex MZM

Here we show that when the type-II adatom under the STM tip in Fig.3 of the main text is moved to another location about 1 nm away (Supplementary Fig. 9a), the transition from the YSR states into a ZBP as shown in Figs. 3b-d at the original location is replicated (Supplementary Fig. 9b). About 27.3% of the type-II Fe adatoms studied by the tip-approaching measurements exhibit the transition from the YSR states to the zero energy bound state tied to the ZBP. This phenomenon should not be induced by the saturation of the exchange coupling strength during the tip approaching, in which case the final energy value should not remain at zero energy frequently. There can be several reasons for the rest of the type-II Fe adatoms not to produce a topological vortex with an MZM. The approaching tip in these

cases may be unsuccessful at nudging the adatoms to the high-symmetry adsorption sites, and/or the spin-orbit exchange interaction has not been increased sufficiently for the nucleation of the QAV. These type-II Fe adatoms may be located in the nontopological surface regions where the topological surface states are absent locally, which is in-line with the observation that only about 20% of the field-induced vortices show the MZM on the same sample surface[1, 6]. Moreover, based on the QAV theory[3], the nucleation of an anomalous vortex at one Fe adatom on the surface is accompanied by that of an anomalous antivortex at another Fe adatom, such that the magnetic flux lines are continuous. While experimental test for this would require multiple STM/S tips in the future, we note that the type-II Fe adatoms displaying the YSR states to vortex MZM transition tend to be located in regions with other Fe adatoms, which can be more favorable for the nucleation of a quantum anomalous vortex-antivortex pair.

SUPPLEMENTARY NOTE 8: Coexistence of MZMs in the QAV and the field-induced vortex

We also observe a field-induced vortex hosting an MZM in an external magnetic field of 1T, which coexists with the MZM in the QAV nucleated at the type-I Fe adatom. The zero-energy dI/dV map (Supplementary Fig. 10a) shows that the field-induced vortex core center is about 7 nm away from the Fe adatom, which is larger than the ones studied in Fig. 4c and 4h of the main text. The intensity plot of the dI/dV spectra (Supplementary Fig. 10a) along the line-cut across both the field-induced vortex and the QAV clearly reveals two ZBPs localized separately in the cores of both vortices. When the external magnetic field is switched off, the field-induced vortex disappears while the QAV at the adatom remains in the zero-energy map (Supplementary Fig. 10c). The dI/dV spectra along the same line-cut show that the hard superconducting gap recovers where the field-induced vortex was located, but the robust ZBP in the QAV nucleated at the Fe adatom. The results indicate that this surface area is in the topological region and there is an MZM in the field-induced vortex in Fig. 4c and Fig. 4h of the main text. These findings support the observation of hybridization between the two MZMs inside

the Fe adatom induced topological QAV and the field-induced topological Abrikosov vortex presented in Fig. 4 of the main text.

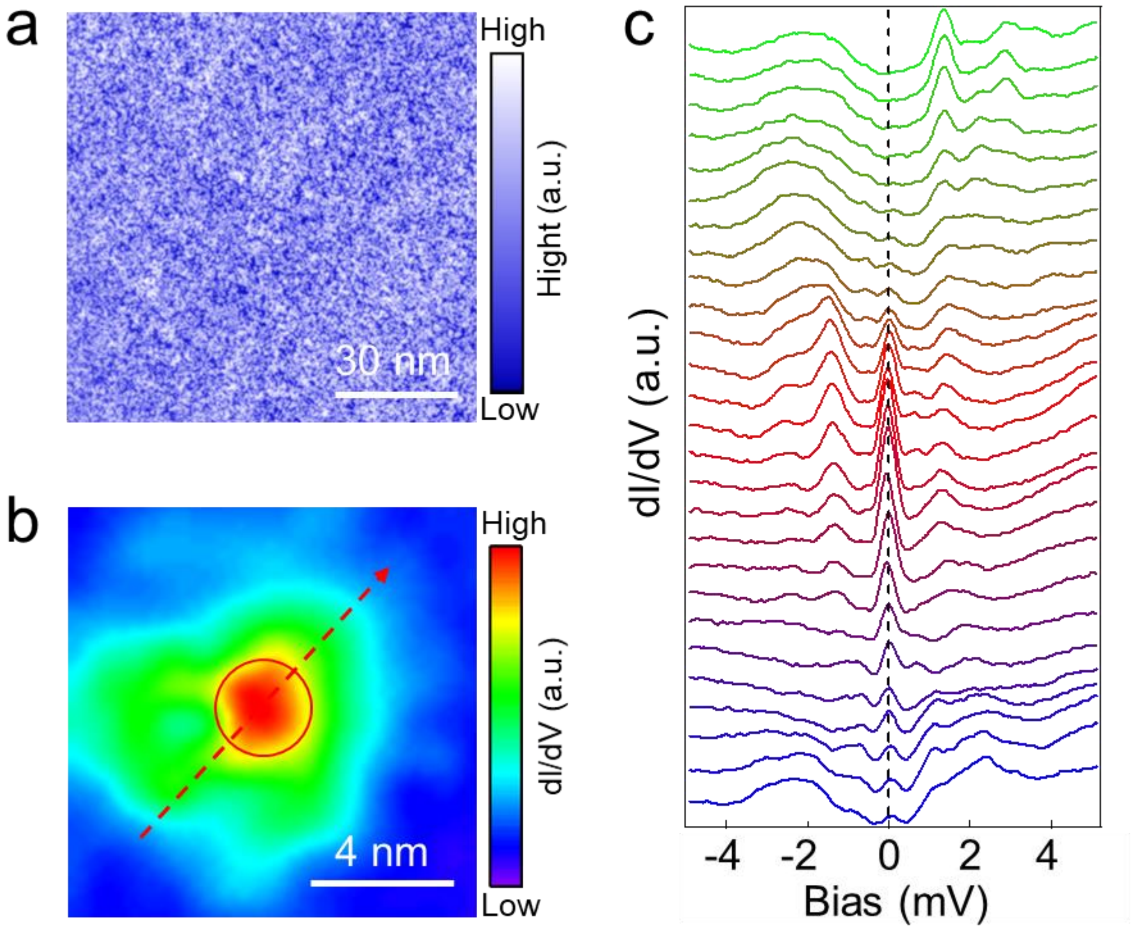

Supplementary Figure 1. MZM in a field-induced vortex detected in $FeTe_{0.55}Se_{0.45}$. a, A STM image (V_s = -10 mV, I_t = 100 pA) of $FeTe_{0.55}Se_{0.45}$ before depositing Fe adatoms. There is no interstitial iron atom. **b**, A zero-energy dI/dV map (V_s = -10 mV, I_t = 100 pA) of a field-induced vortex with a MZM in the same area of **a**. The field-induced vortex center is marked by the red circle in **a** and **b**. **c**, dI/dV spectra (V_s = -10 mV, I_t = 200 pA) along the line-cut indicated in **b**, showing a sharp ZBP that does not split or shift with the changes of spatial position, consistent with previous observation of vortex MZMs in the same material.

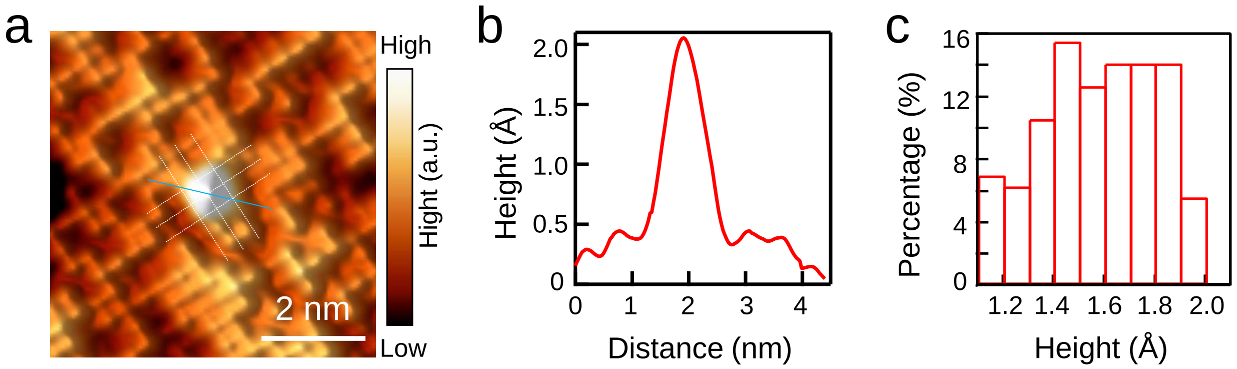

Supplementary Figure 2. Characterization of deposited single Fe adatom on $FeTe_{0.55}Se_{0.45}$ surface. **a**, A high-resolution STM image (V_s = -10 mV, I_t = 100 pA) showing a single Fe adatom located at the C_4 symmetric site. The C_4 symmetric site is highlighted by the vertices of the white dashed grid. **b**, A line-profile (V_s = -10 mV, I_t = 100 pA) along the light blue line in **a**, showing that the single Fe adatom is 1.61 Å in height and 1.5 nm in width (defined as the peak width at half maximum). **c**, A histogram showing the statistics of the height distribution of hundreds of Fe adatoms. The height varies from 1.1 Å to 2.0 Å.

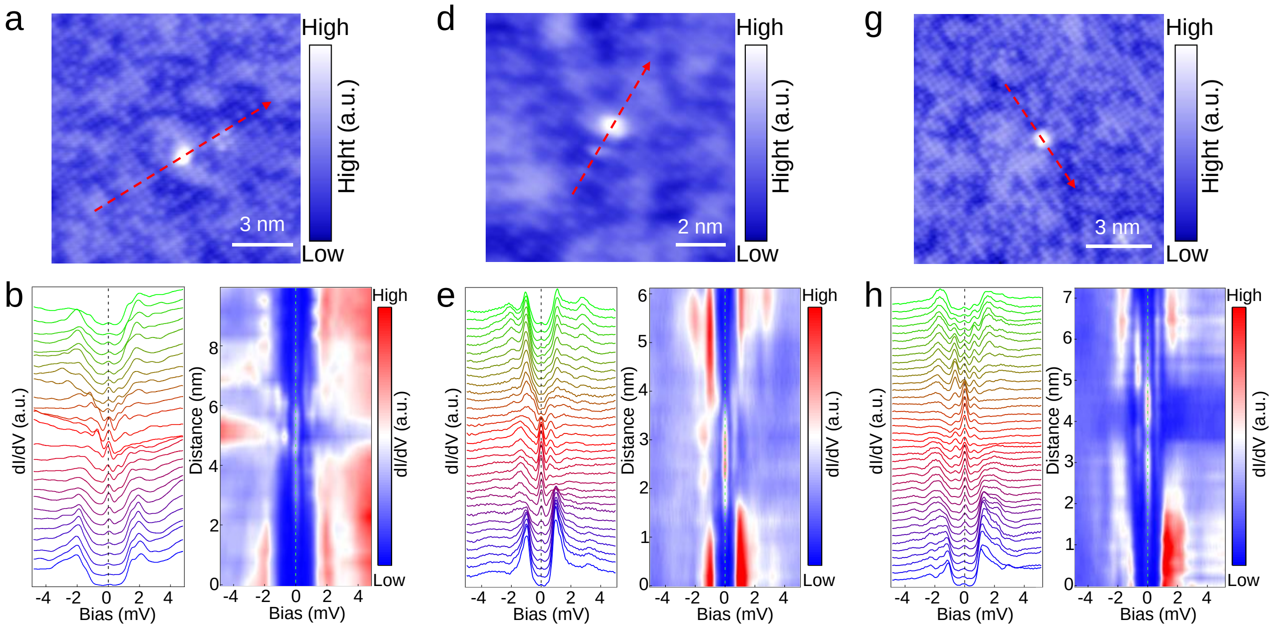

c

	E_n (meV)	E_n / ΔE
E_3	1.88	3.13
E_2	1.15	1.91
E_1	0.60	1.00
E_0	0.00	0.00

f

	E_n (meV)	E_n / ΔE
E_3	1.90	3.22
E_2	1.18	2.00
E_1	0.59	1.00
E_0	0.00	0.00

i

	E_n (meV)	E_n / ΔE
E_3	2.07	3.09
E_2	1.29	1.93
E_1	0.67	1.00
E_0	0.00	0.00

Supplementary Figure 3. Integer quantized in-gap states on type-I Fe adatoms. a, **d** and **g**, Atomic-resolution topographic images (V_s = -10 mV, I_t = 100 pA) of type-I Fe adatoms. **b**, **e** and **h**, Waterfall and intensity plots of dI/dV spectra (V_s = -10 mV, I_t = 200 pA) measured along the line-cut indicated correspondingly in **a**, **d** and **g** across the three adatoms, showing a sharp ZBP coexisting with several pairs of discrete in-gap states. **c**, **f** and **i**, Lists of the energy positions of the in-gap states corresponding to **b**, **e** and **h**, respectively, showing near integer quantized energy level spacing which is the hallmark of the CdGM vortex core states of the topological vortex.

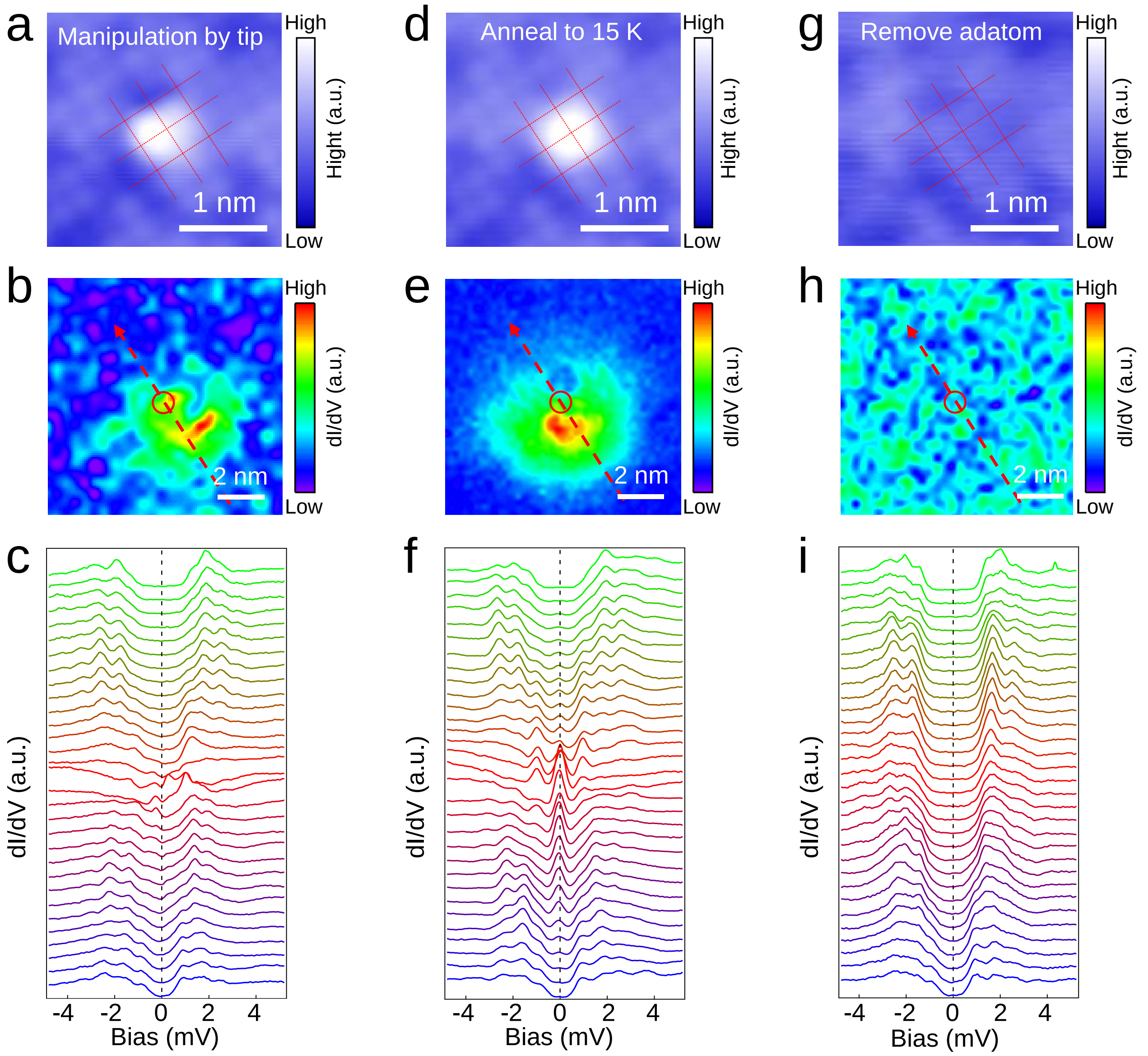

Supplementary Figure 4. Manipulation of a type-I Fe adatom. a, A STM image (V_s = -10 mV, I_t = 100 pA) showing that the Fe adatom in Fig. 2 of the main text has been moved off the C_4 symmetric center by the STM tip. **b**, A zero-energy dI/dV map (V_s = -10 mV, I_t = 100 pA) around the Fe adatoms in **a**. The pattern is significantly different from that before the manipulation shown in Fig. 2b of the main text. **c**, dI/dV spectra (V_s = -10 mV, I_t = 200 pA) along the line-cut indicated in **b** showing that the ZBP disappears and other in-gap states emerge. **d**, A STM image (V_s = -10 mV, I_t = 100 pA) showing that the Fe adatom moves back to the center of four Te adatoms by *in situ* annealing the sample and cooling back to 0.4 K. **e**, A zero-energy dI/dV map (V_s = -10 mV, I_t = 100 pA) around the Fe adatoms in **d**. The pattern recovers the spatial distribution of the zero-mode before the manipulation (Fig. 2b of the main text). **f**, dI/dV line-cut spectra (V_s = -10 mV, I_t = 200 pA) along the red dashed

arrow in **e** showing that the ZBP reappears. **g**, A topographic image (V_s = -10 mV, I_t = 100 pA) showing the removal of the Fe adatom from the field of view by the STM tip. **h**, The zero-energy dI/dV map (V_s = -10 mV, I_t = 100 pA) around the Fe adatoms in **g** shows that there are no low energy bound states after removing the Fe adatom. **i**, dI/dV line-cut spectra (V_s = -10 mV, I_t = 200 pA) along the red dashed arrow in **h**, showing the evolution of a hard superconducting gap.

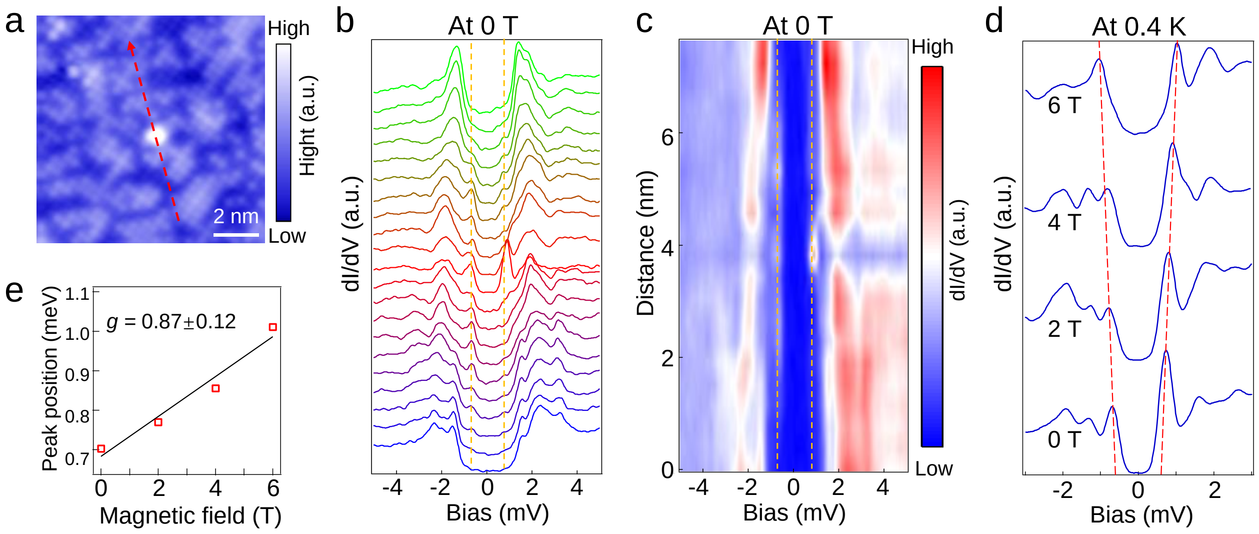

Supplementary Figure 5. YSR states on type-II Fe adatoms and Zeeman splitting. a, An atomic-resolution topographic image (V_s = -10 mV, I_t = 100 pA) showing a single type-II Fe atom. **b** and **c**, dI/dV spectra (V_s = -10 mV, I_t = 200 pA) and the corresponding intensity plot (V_s = -10 mV, I_t = 200 pA) along the line-cut indicated by the red dashed and arrowed line in **a**. The dashed lines highlight the emergence of the pair of YSR states near the Fe adatom. **d**, Magnetic field dependence of the dI/dV spectra (V_s = -10 mV, I_t = 200 pA) acquired at the adatom. Red dashed lines trace out of the spin-polarized Zeeman splitting of the YSR states. **e**, A linear fit of the YSR state energy as a function of magnetic field, yielding an estimated *g*-factor ~ 0.87.

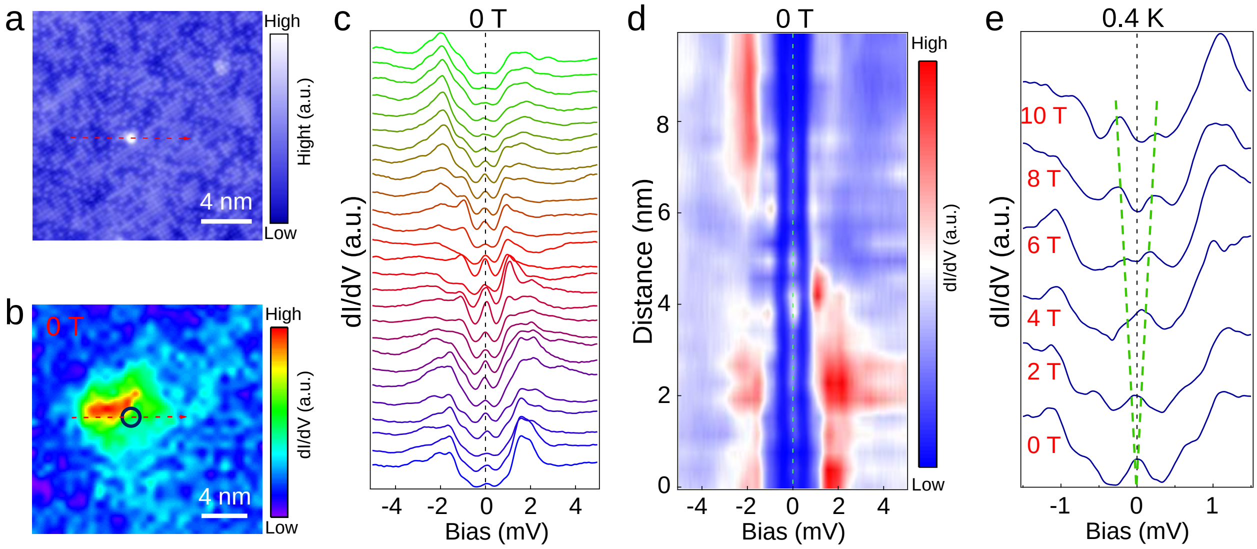

Supplementary Figure 6. Near-zero energy YSR states and Zeeman splitting. **a**, An atomic-resolution STM image (V_s = -10 mV, I_t = 100 pA) of a type-II Fe adatom. **b**, A zero-energy dI/dV map (V_s = -10 mV, I_t = 100 pA) of the same area in **a**. **c** and **d**, dI/dV spectra (V_s = -10 mV, I_t = 200 pA) and the corresponding intensity plot along the line-cut indicated by the red dashed arrow in **a**, showing nearly degenerate YSR states close to zero energy highlighted by the dashed line. **e**, Magnetic field dependence of the dI/dV spectra (V_s = -10 mV, I_t = 200 pA) acquired at the adatom site (black circle in **b**). The green dashed lines trace out of the Zeeman splitting of the YSR states.

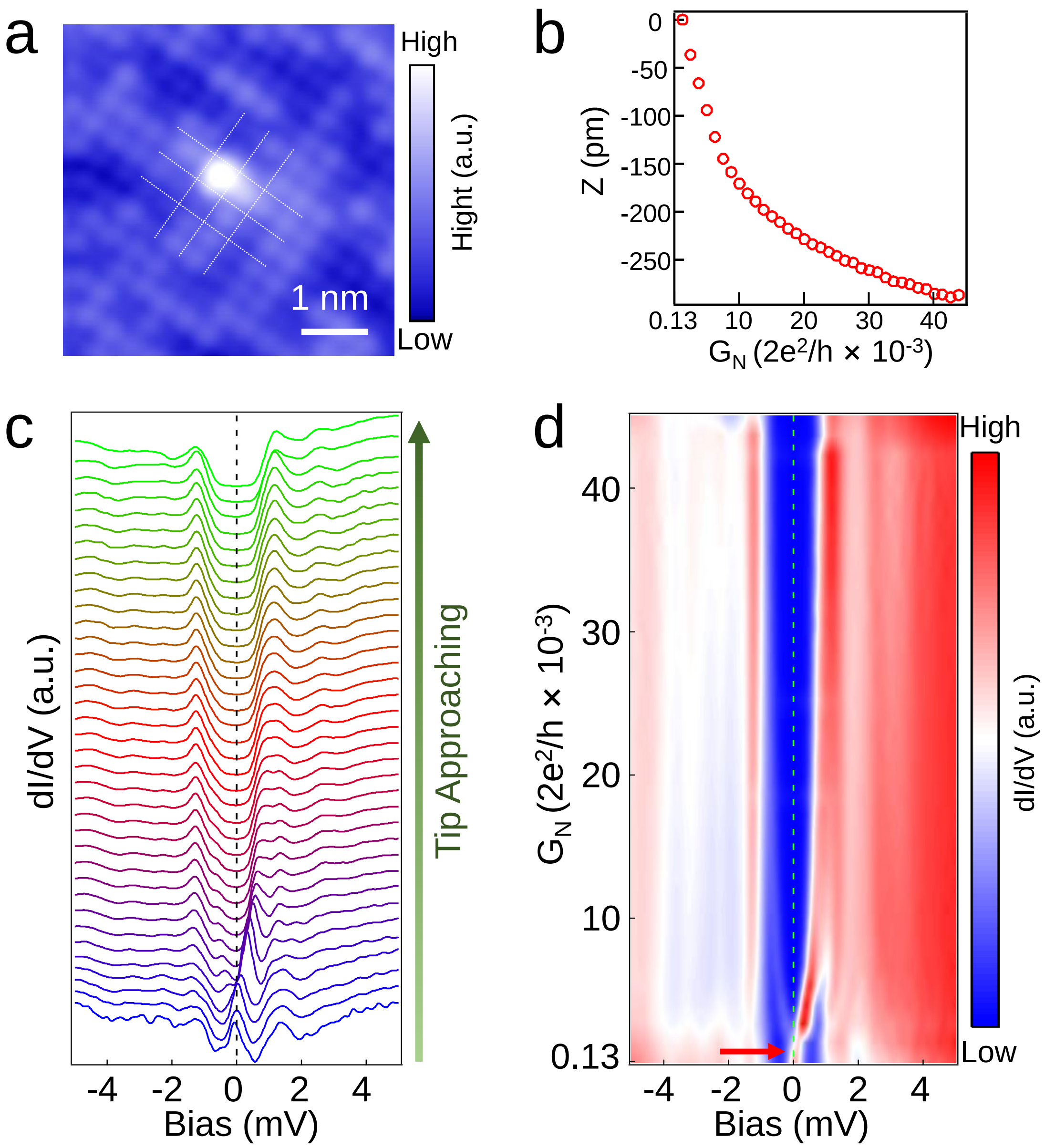

Supplementary Figure 7. Modulating YSR states with approaching STM tip at a type-II Fe adatom. a, A high-resolution STM image (V_s = -10 mV, I_t = 100 pA) showing a single type II Fe adatom located away from the C_4 symmetric site (on the vertices of the white dashed grids). **b**, The tip-sample distance offset z versus tunnel-barrier conductance G_N as the tip approaches the adatom. The z-offset decreases smoothly with increasing barrier conductance G_N. **c** and **d**, The normalized dI/dV spectra (V_s = -10 mV, I_t = 200 pA) and the corresponding intensity plot under different tunnel-barrier conductance in zero applied magnetic field, showing the shift of YSR states. The red arrow indicates the quantum transition point of the YSR states.

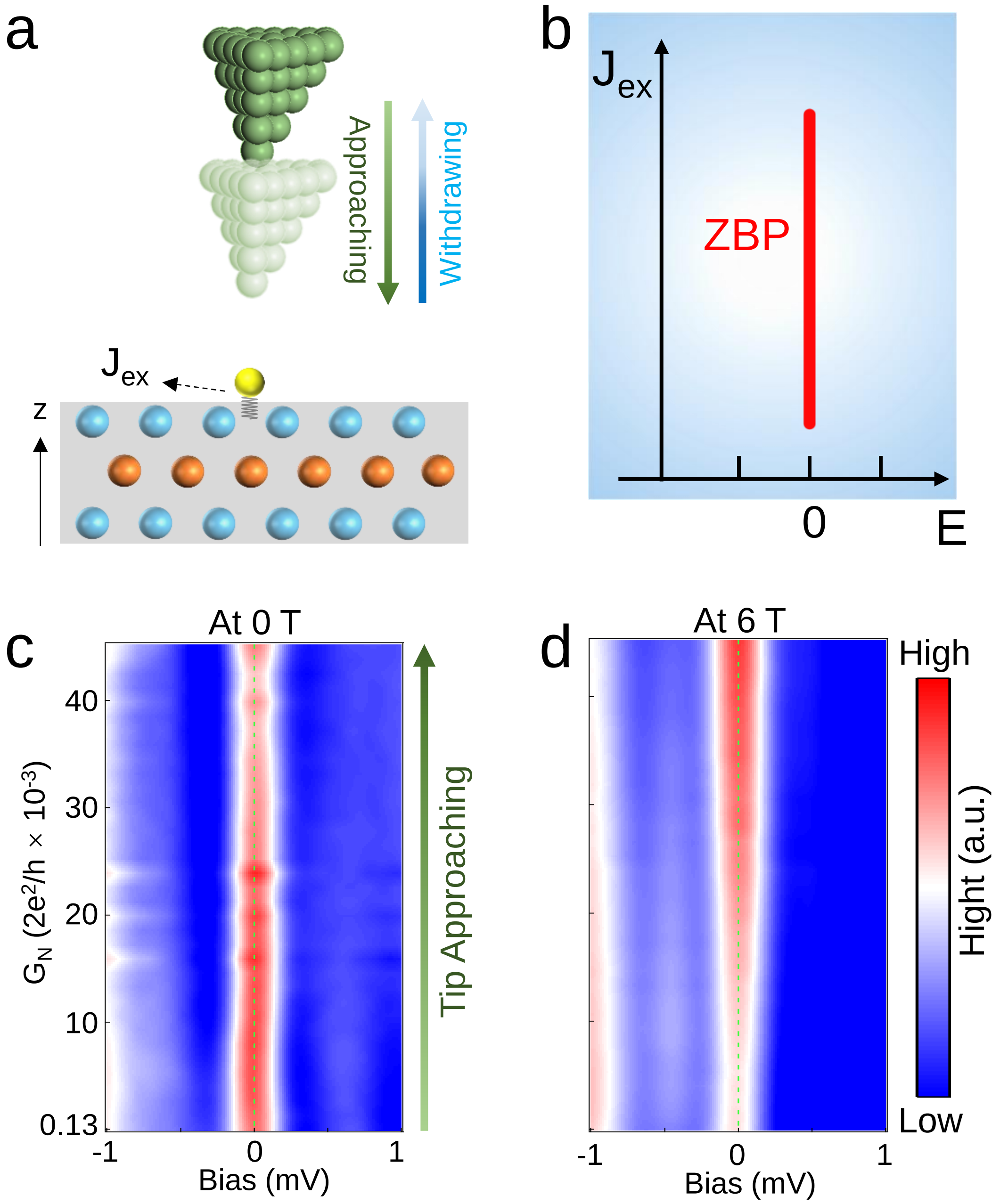

Supplementary Figure 8. Robustness of ZBP under approaching STM tip at a type-I Fe adatom. **a**, Schematics illustrating an approaching STM tip on top of a type-I Fe adatom. The Fe adatom is located at the high-symmetry site and is close enough to the surface. The exchange coupling is strong enough to produce a ZBP without approaching tip. **b**, Schematic diagram showing the robustness of the ZBP at type-I Fe adatoms against increasing exchange interaction (J_{ex}). **c** and **d**, Intensity plots of the dI/dV spectra, showing the evolution of the robust ZBP with increasing tunnel barrier conductance at 0 T (**c**) and 6 T (**d**).

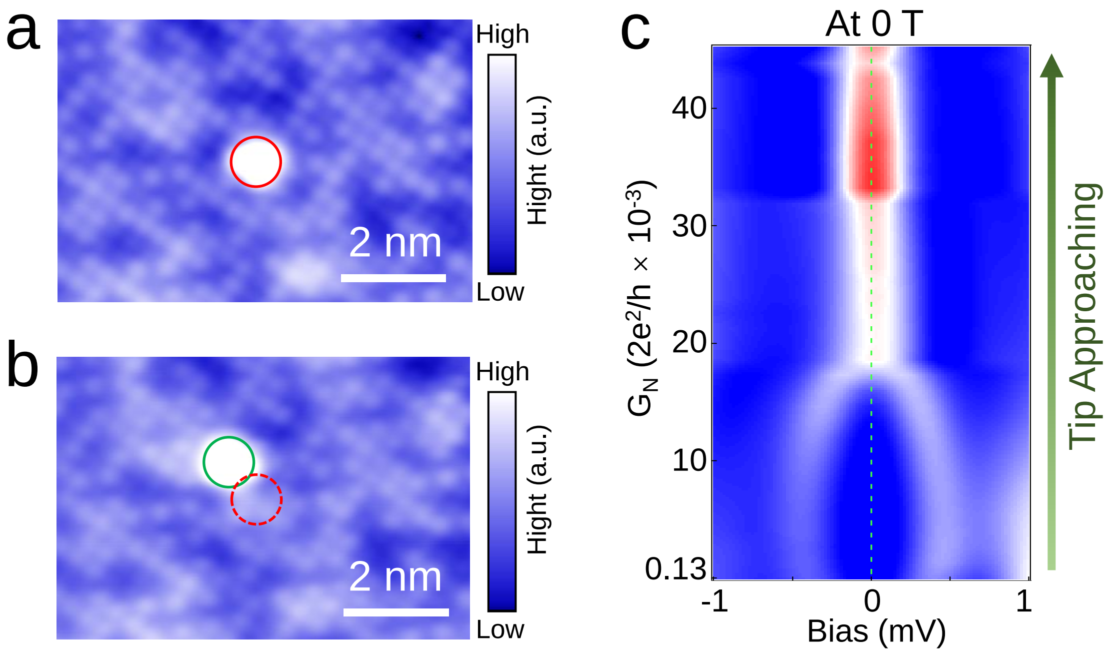

Supplementary Figure 9. Replicable transition between YSR states and a vortex MZM. a and **b**, STM images (V_s = -10 mV, I_t = 100 pA) showing the position of the Fe adatom before (red circle) and after (green circle) the STM manipulation. The dI/dV spectra in Figs. 3c-f of the main text correspond to the adatom located in the red circle. **c**, Intensity plot of dI/dV spectral evolution with tunnel barrier conductance at the Fe adatom located in the green dotted circle in **b**. The transition between the YSR states and the vortex MZM represented by the ZBP observed in Fig. 3 of the main text is reproduced at the new location.

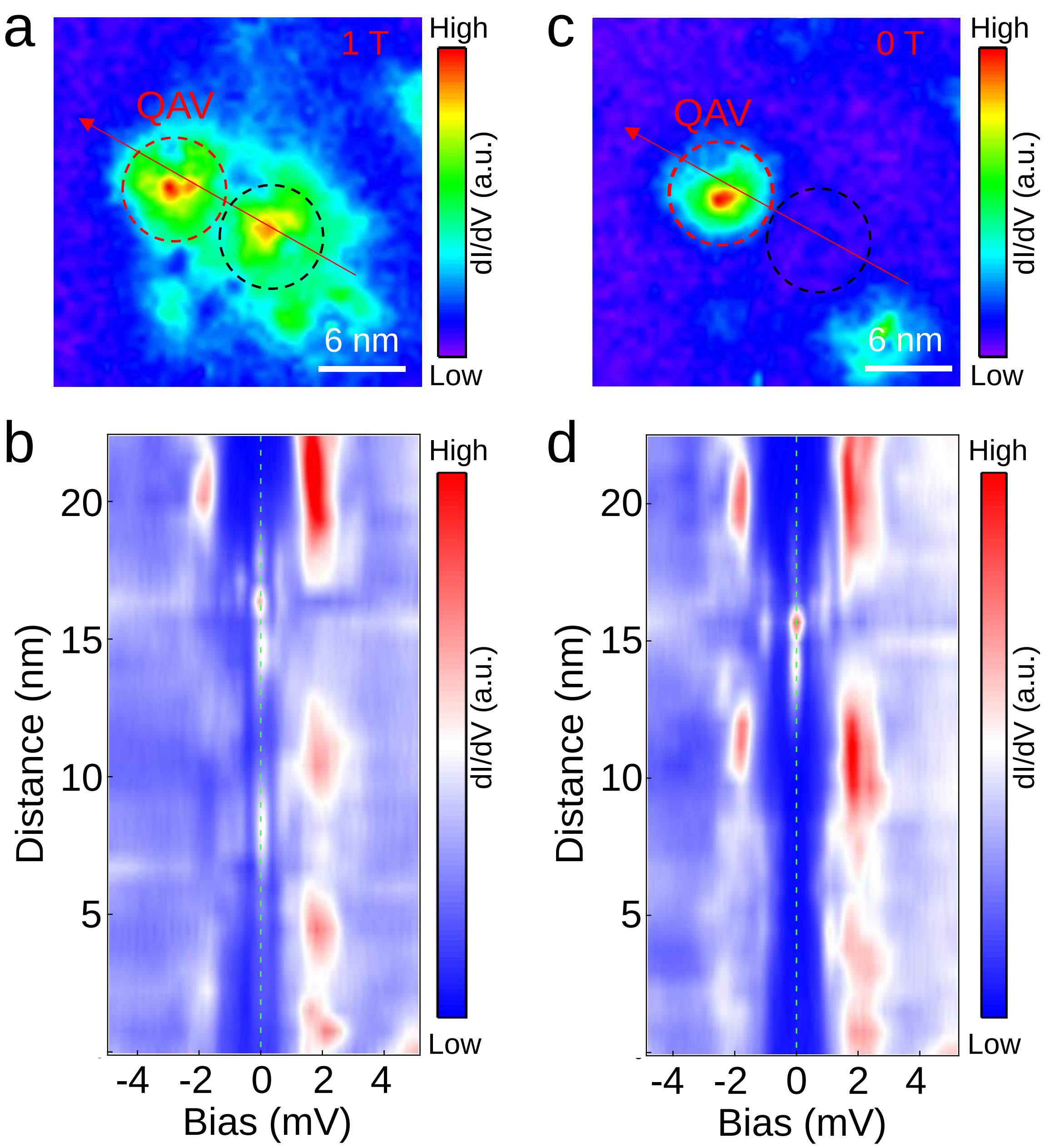

Supplementary Figure 10. Coexistence of MZMs in a QAV and a field-induced vortex. a, The zero-energy dI/dV map (V_s = -10 mV, I_t = 100 pA) in an applied field of 1 T around a type-I Fe atom. The QAV nucleated at the adatom (red dashed circle) is the same one shown in Fig. 4 of the main text. The center of the field-induced topological vortex (black dashed circle) is about 7 nm away from the center of the QAV. **b**, Intensity plot of dI/dV spectra (V_s = -10 mV, I_t = 200 pA) along the red arrow in **a**, showing two ZBPs localized separately in the cores of both vortices. **c**, The zero-energy dI/dV map (V_s = -10 mV, I_t = 100 pA) before applying the magnetic field in the same area. **d**, Intensity plot

of dI/dV spectra (V_s = -10 mV, I_t = 200 pA) along the red arrow in **c**, showing the hard superconducting gap in the region occupied by the field-induced vortex (black dashed circle).

References:

1. Kong, L. et al. Half-integer level shift of vortex bound states in an iron-based superconductor. *Nat Phys* **15**, 1181-1187 (2019).
2. Yin, J. X. et al. Observation of a robust zero-energy bound state in iron-based superconductor Fe(Te,Se). *Nat Phys* **11**, 543-546 (2015).
3. Jiang, K., Dai, X. & Wang, Z. Q. Quantum anomalous vortex and Majorana zero mode in iron-based superconductor Fe(Te,Se). *Phys Rev X* **9**, 011033 (2019).
4. Farinacci, L. et al. Tuning the coupling of an individual magnetic impurity to a superconductor: quantum phase transition and transport. *Phys Rev Lett* **121**, 196803 (2018).
5. Malavolti, L. et al. Tunable spin–superconductor coupling of spin 1/2 vanadyl phthalocyanine molecules. *Nano Lett* **18**, 7955-7961 (2018).
6. Wang, D. F. et al. Evidence for Majorana bound states in an iron-based superconductor. *Science* **362**, 333-335 (2018).
